# Supplementary material for: Recombinant protein delivery enables modulation of the phototransduction cascade in mouse retina
Source: Cell Mol Life Sci. 2023 Nov 25;80(12):371. doi: 10.1007/s00018-023-05022-0 (PMC10673981; doi:10.1007/s00018-023-05022-0)
Supplement: Supplementary file 1 — Supplementary file1 (PDF 5196 KB) [file 18_2023_5022_MOESM1_ESM.pdf]

Supplementary Information

Recombinant protein delivery enables modulation of the phototransduction cascade in mouse retina

Sabrina Asteriti, Valerio Marino, Anna Avesani, Amedeo Biasi, Giuditta Dal Cortivo, Lorenzo Cangiano and Daniele Dell'Orco

This PDF file includes:

Figures S1 to S9
Table S1
Captions for Movies S1 to S5

Other Supplementary Materials for this manuscript include the following:

Movies S1 to S5

Figure S1

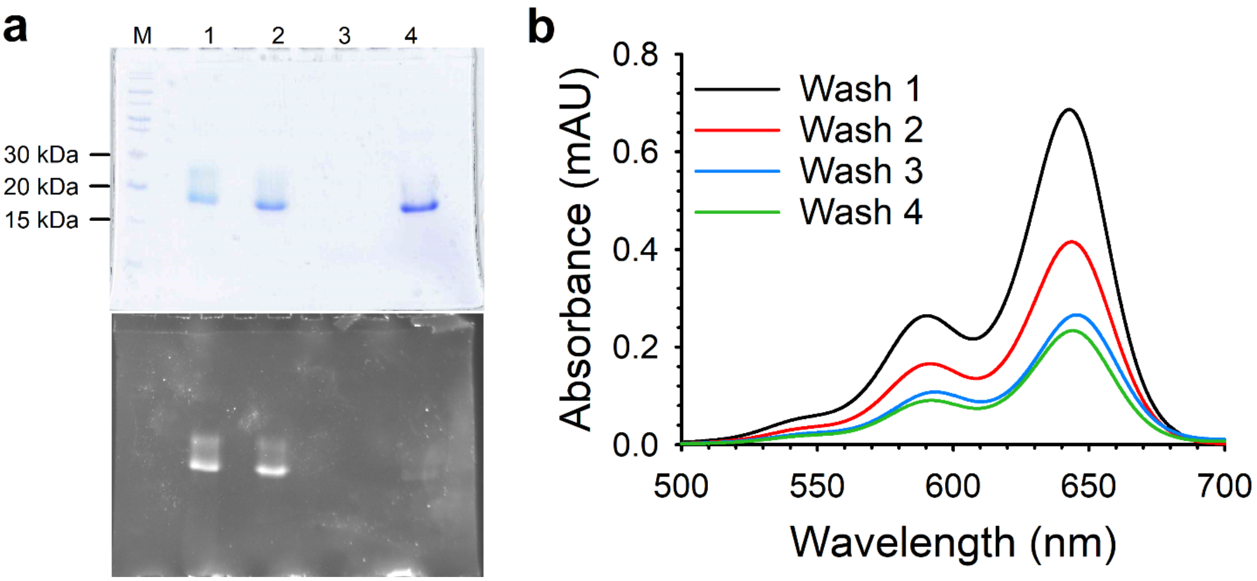

Assessment of the conjugation of GCAP1 with CF640R and removal of free dye. **a)** 15% SDS-PAGE of GCAP1 before and after conjugation with CF640R and after encapsulation in LPs. Lanes: M) marker, 1) LP-GCAP1^{CF640R}, 2) free-GCAP1^{CF640R}, 3) free-CF640R, 4) WT-GCAP1, stained with Coomassie blue (top panel) and upon excitation at 580 nm (bottom panel). **b)** Representative absorption spectra of the flowthrough of the 4 sequential washing steps to remove unconjugated dye.

Figure S2

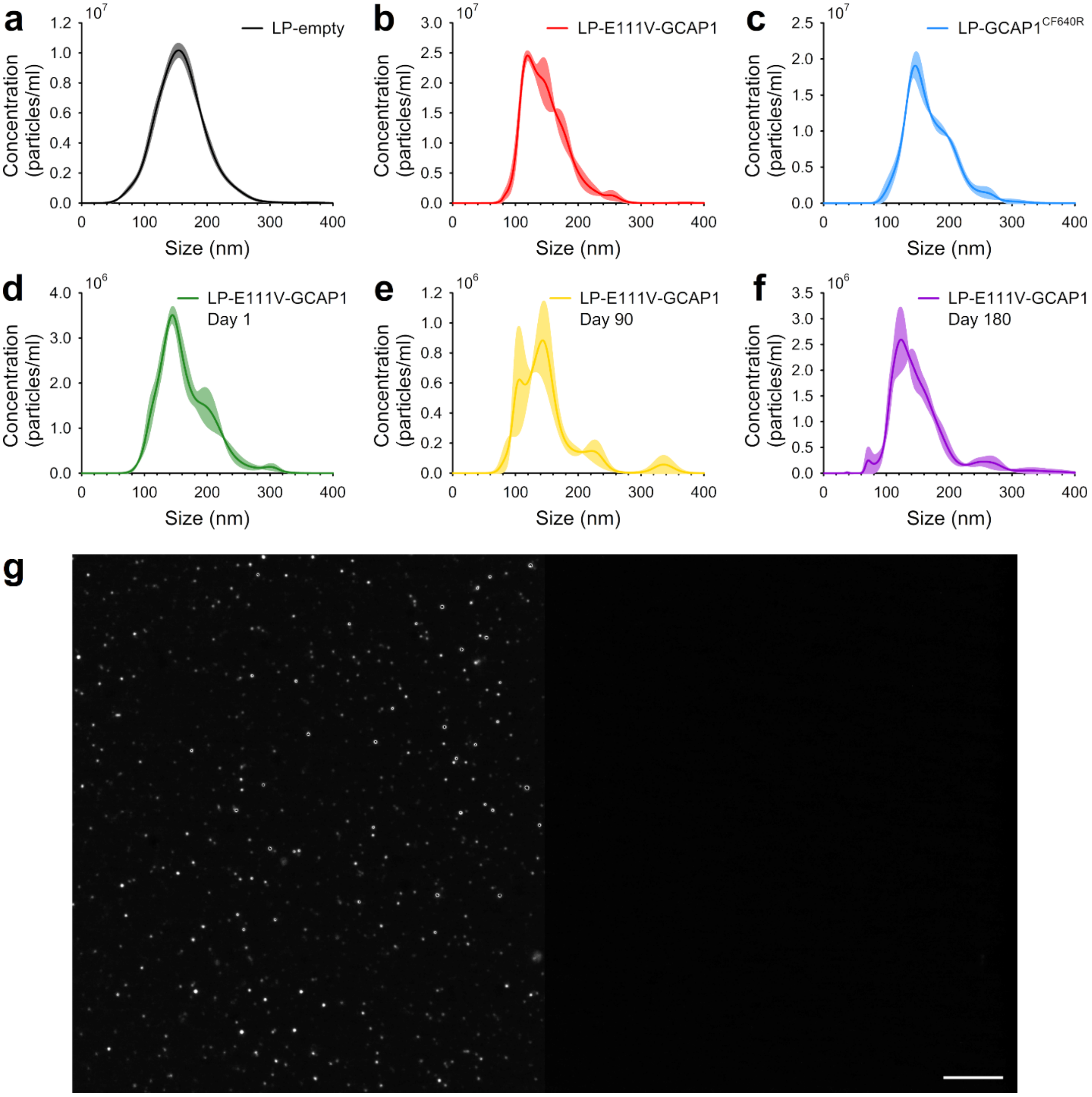

Representative profiles of the size of **a)** 5.1 nM LP-empty (black), **b)** 4.3 nM LP-E111V-GCAP1 (red), **c)** 4.6 nM LP-GCAP1^{CF640R} (blue) estimated by NTA. Monitoring of the size of ~2.9 nM (**Table S1**) LP-E111V-GCAP1 after **d)** 1 (green), **e)** 90 (yellow) and **f)** 180 days (violet). Each plot represents the average of 3 independent measurements, standard errors are displayed as a

lighter shade of the color of each trace. Concentrations refer to the stock solutions, before dilutions required for NTA analysis. **g**) LP-CF640R appear as point-like (diffraction limited) fluorescence when suspended in agarose gel (left field) while empty LPs do not (right field). The two fields were acquired, processed, and displayed with identical parameters. Scale bar 10 µm.

Figure S3

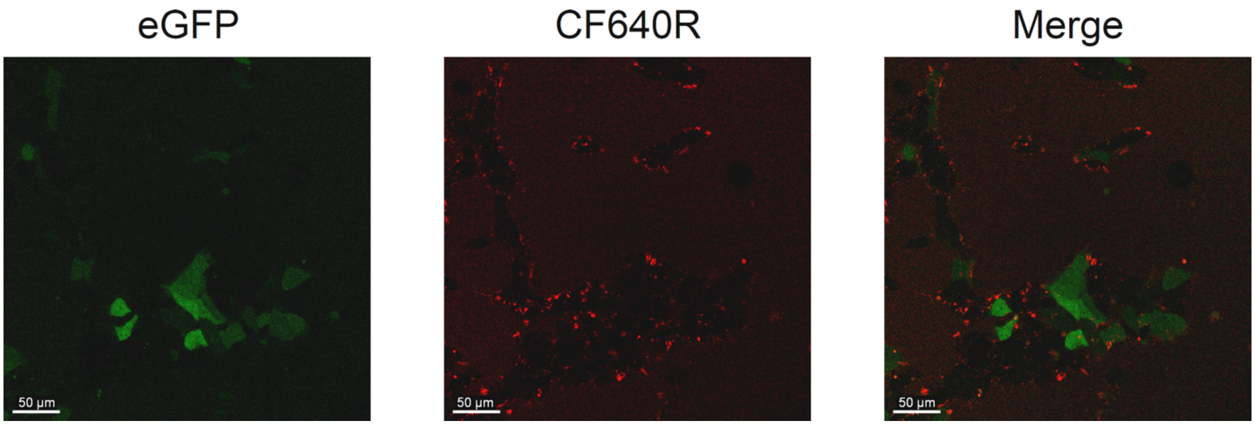

Representative images of cGFP cell line after 24 h incubation with 100 µl of 104 µM free-GCAP1^{CF640R} after replacing cell medium with FluoroBrite DMEM. Left panel shows eGFP fluorescence, center panel shows CF640R fluorescence, left panel shows merged fluorescence.

Figure S4

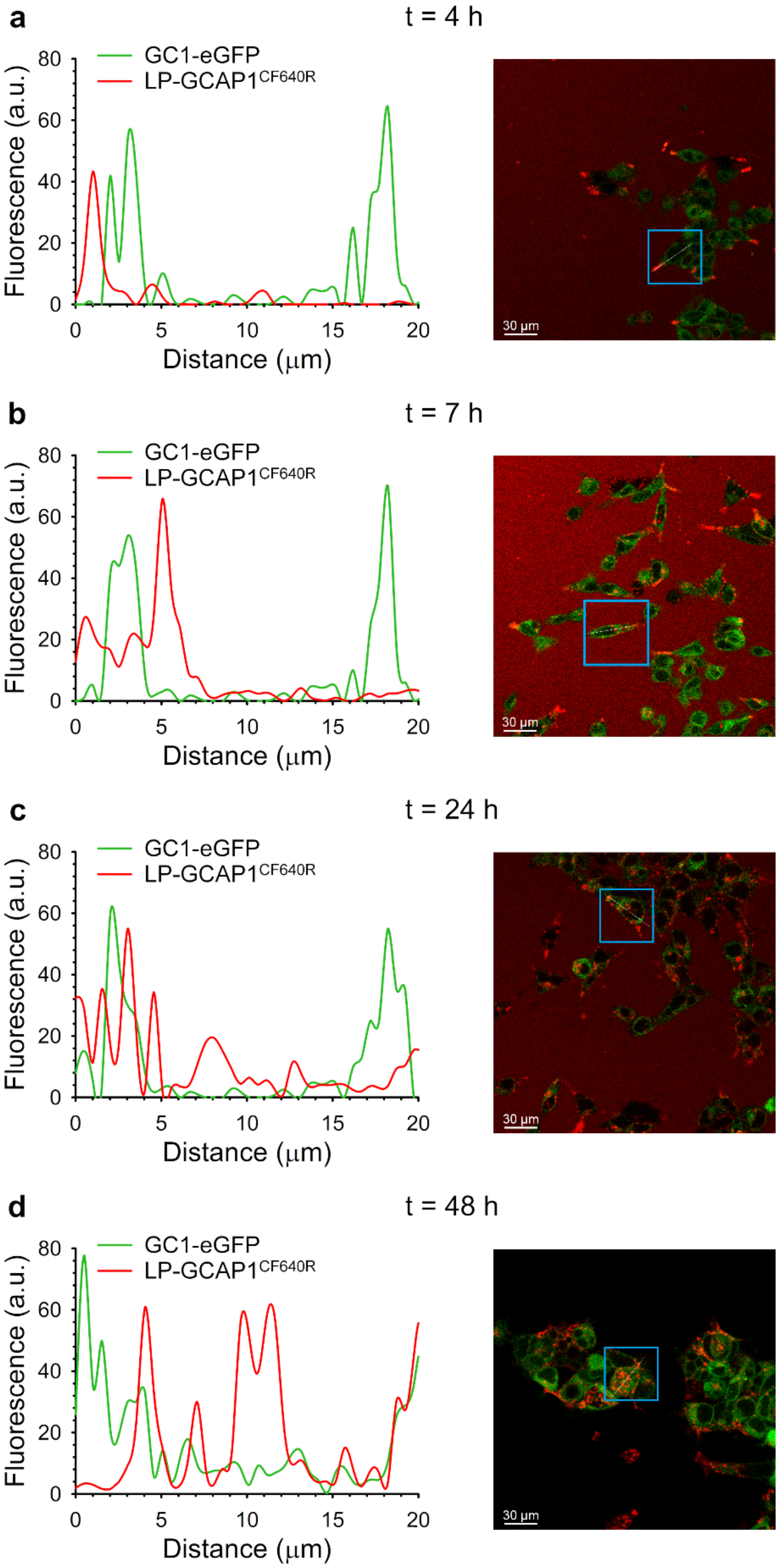
a
t = 4 h
GC1-eGFP
LP-GCAP1CF640R
Fluorescence (a.u.)
Distance (μm)
30 μm
b
t = 7 h
GC1-eGFP
LP-GCAP1CF640R
Fluorescence (a.u.)
Distance (μm)
30 μm
c
t = 24 h
GC1-eGFP
LP-GCAP1CF640R
Fluorescence (a.u.)
Distance (μm)
30 μm
d
t = 48 h
GC1-eGFP
LP-GCAP1CF640R
Fluorescence (a.u.)
Distance (μm)
30 μm

Representative (n= 6) fluorescence intensity profiles (left column) of live cell imaging (right column) of mGFP cell line (green) after **a)** 4 h, **b)** 7 h, **c)** 24 h and **d)** 48 h incubation with 100 µl of 4.6 nM LP-GCAP1^{CF640R} (containing 27.4 µM GCAP1^{CF640R} in the aqueous core, red). Fluorescence profiles were collected on the same z- plane as in **Fig. 3c**. Representative profiles refer to the cell framed in blue along the white line.

Figure S5

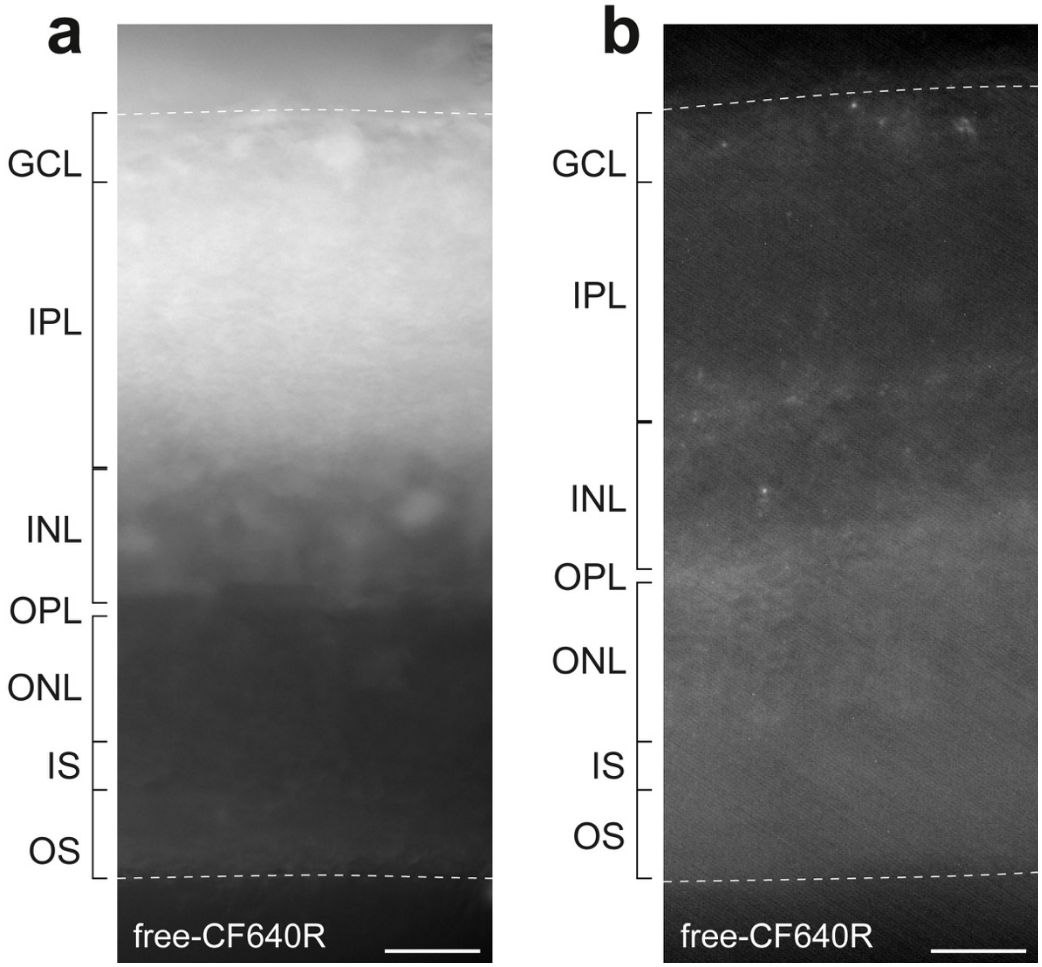

Biodistribution of free CF640R in mouse retinal slices following either **a)** *ex vivo* incubation with 20 µl of 140 µM CF640R in 2 ml Ames' medium (3.5 h at 37°C), or **b)** intravitreal injection of 2 µl of the same stock solution (24 h). Scale bars 25 µm.

Figure S6

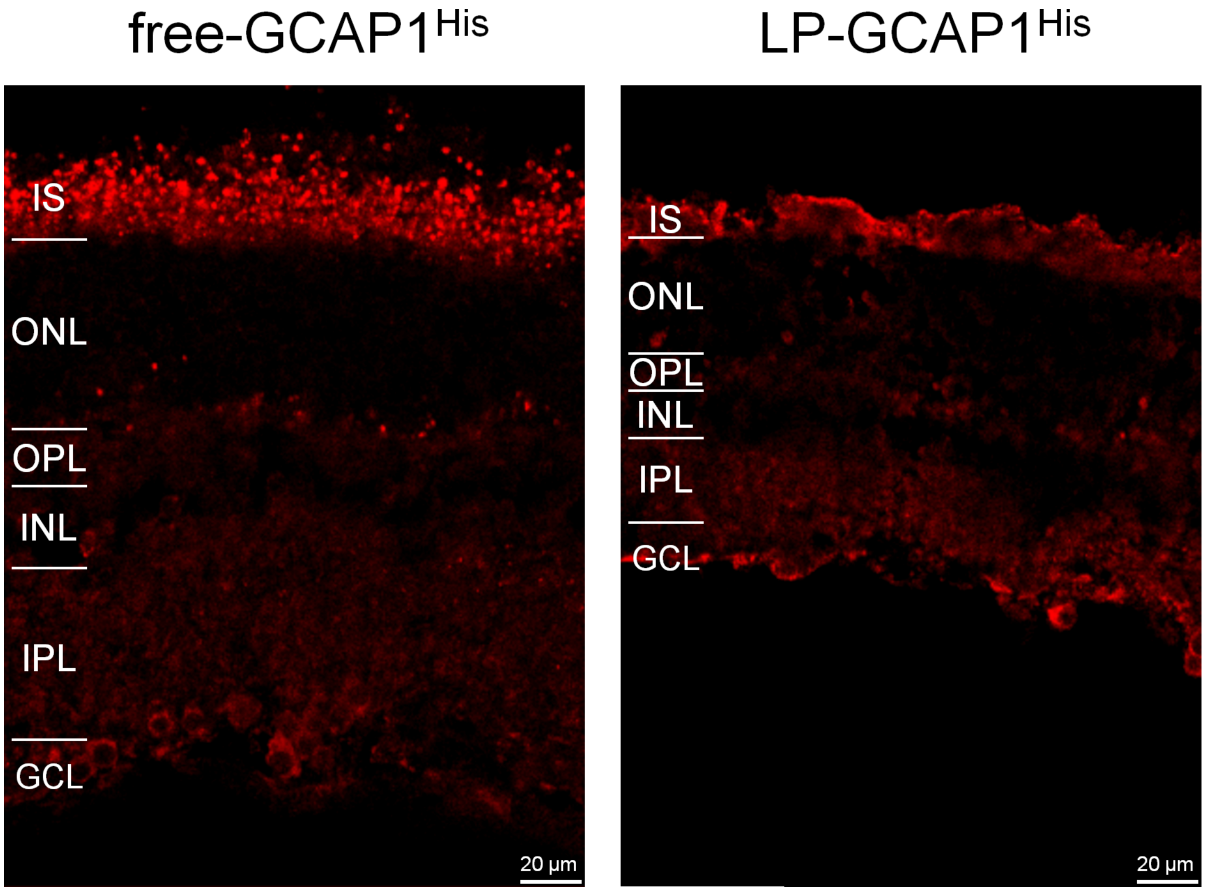

Representative central plane of Z-stack of retinal cryosections after 24 h incubation with 180 µl of 99.6 µM free-GCAP1^{His} (left panel) and 4.5 nM LP-GCAP1^{His} (right panel). Sections were stained with an anti-His antibody (red) using the same image acquisition and display parameters (compare with Fig. 4 in the main text, where nuclei have been stained). Note the presence of more diffused and speckled signals in the case of delivered free-protein, and the prevalence of diffused signal in the case of LP-encapsulated protein at this time frame.

Figure S7

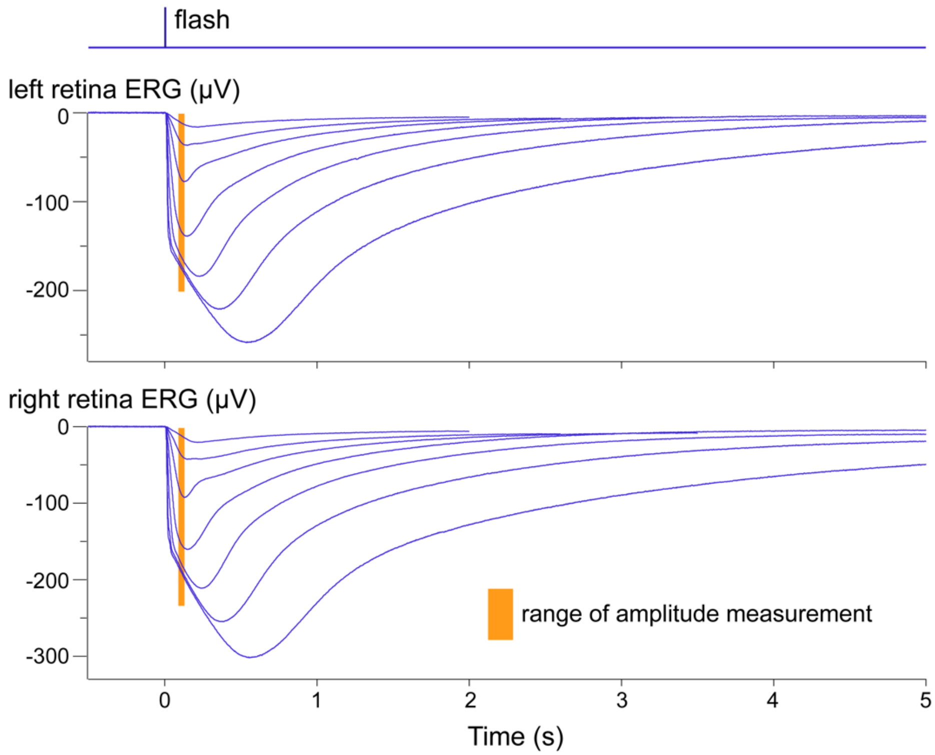

Examples of concurrently recorded *ex vivo* ERG from the two retinas of a mouse. Each trace is the average of several flash responses, with an entire flash family being delivered every 15 min. Flash strengths (ph/µm^2)|no. of repetitions: 3.98|12, 8.27|10, 18.9|8, 50.5|6, 151|6, 510|4, 1660|3. Light sensitivity (i_{50}) was estimated by fitting a Hill function to the response amplitudes measured in the range 90–130 ms after the flash (orange areas). Kinetics (TTP@i_{50}) was estimated as the time to peak of the hypothetical response at i_{50}. Left retina: i_{50} = 23.2 ph/µm^2; TTP@i_{50} = 136 ms. Right retina: i_{50} = 20.7 ph/µm^2; TTP@i_{50} = 136 ms.

Figure S8

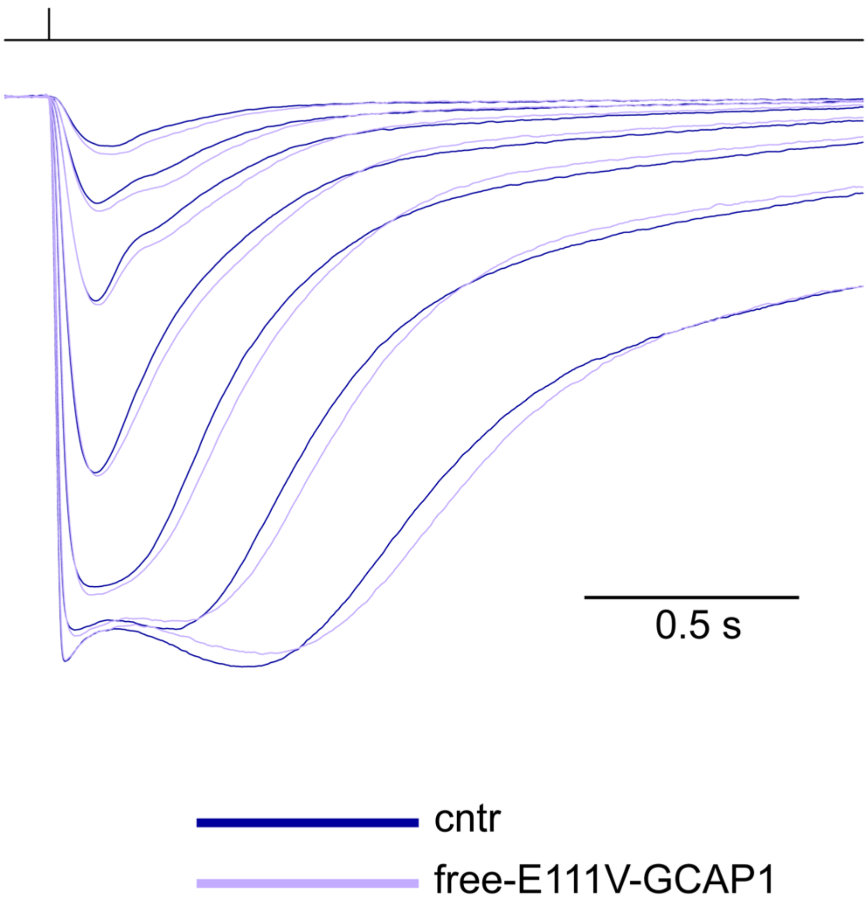

Example of the effect of free-E111V-GCAP1 in slowing scotopic flash response kinetics. $BaCl_2$ (50 µM) was first injected in the 2 ml well followed, after 45 min, by injection of 100 µl free-E111V-GCAP1. The control flash family (blue traces) was recorded just before protein injection, while the treated one (purple traces) after 37 min. Each trace is the average of several responses and the data shown here is from one of the experiments that also contributed to fig. 6h. Note that the effect of the free protein on response kinetics is underestimated in this figure, since the untreated retina in this pair showed a slight acceleration of kinetics over the same period. This highlights the importance of analyzing the effect of the injected formulations by normalizing 'treated' over 'control' retinas, as done in Fig. 6 (see also the Methods section).

Figure S9

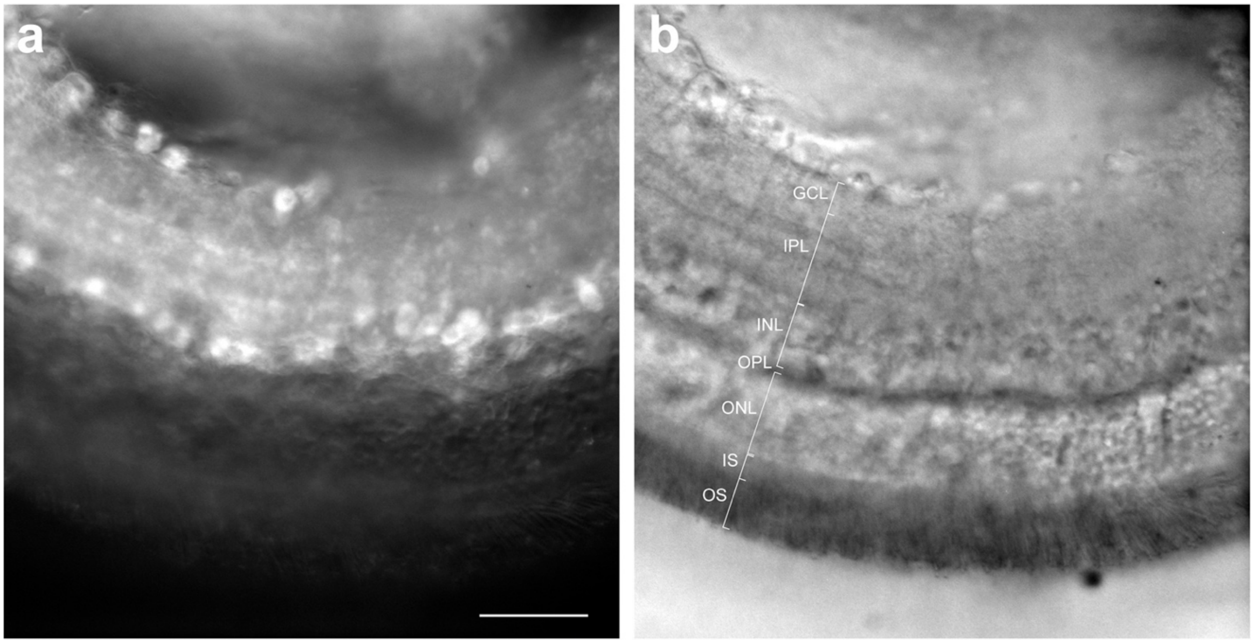

Method of identification of layer boundaries in live slices from *ex vivo* incubated and intravitreally injected retinas. This example shows a slice from a retina incubated *ex vivo* with LP-GCAP1^{CF640R}. **a)** Fluorescence image obtained with the widefield microscope (CY5 filterset, epi-illumination). **b)** The same field and focal plane illuminated and imaged in the near infrared (750 nm LED source, trans-illumination): the various retinal layers were readily distinguishable. All slices used in the analyses of **Fig. 3** were similarly imaged both in fluorescence and in the IR to facilitate layer recognition. Scale bar 50 µm.

Table S1

Liposomes	Size (nm)	Concentration (nM)
LP-empty	158.6 ± 1.1	5.1 nM
LP-E111V-GCAP1	153.3 ± 2.9	4.3 nM
LP-GCAP1^{CF640R}	168.7 ± 0.7	4.6 nM
LP-empty (day 1)	149.1 ± 3.0	4.6 nM
LP-empty (day 90)	151.0 ± 5.4	3.2 nM
LP-empty (day 180)	157.4 ± 2.0	4.0 nM
LP-E111V-GCAP1 (day 1)	164.3 ± 0.4	2.9 nM
LP-E111V-GCAP1 (day 90)	152.1 ± 3.4	2.6 nM
LP-E111V-GCAP1 (day 180)	153.7 ± 1.8	3.0 nM
LP-CF640R	160.5 ± 1.2	3.9 nM
LP-GCAP1^{His}	171.3 ± 4.8	4.5 nM

Size, concentration, and stability over 180 days of LPs loaded with different molecules (dissolved in PBS) monitored by NTA. Data refer to the mean ± standard error of 3 technical replicates.

Movie S1

The three-dimensional structure of GCAP1 is shown as light-grey cartoon with the molecular surface in transparency, Ca^{2+}-ions are displayed as green spheres, the sidechains of Lys residues are labelled represented as red sticks with N atoms in blue. The molecular surface of the primary amines belonging to Lys sidechains is shown in blue in transparency.

Movie S2

Live-cell imaging at 6 h of cGFP cell line incubated with 100 µl of 140 µM free-CF640R, snapshots were acquired at a 30 min interval, green fluorescence refers to eGFP, red fluorescence refers to free-CF640R molecules.

Movie S3

Live-cell imaging at 6 h of mGFP cell line incubated with 100 µl of 104 µM free-$GCAP1^{CF640R}$, snapshots were acquired at a 30 min interval, green fluorescence refers to eGFP, red fluorescence refers to free-$GCAP1^{CF640R}$ molecules.

Movie S4

Live-cell imaging at 6 h of cGFP cell line incubated with 100 µl of 104 µM free-$GCAP1^{CF640R}$, snapshots were acquired at a 30 min interval, green fluorescence refers to eGFP, red fluorescence refers to free-$GCAP1^{CF640R}$ molecules.

Movie S5

Live-cell imaging at 24 h of mGFP cell line incubated with 100 µl of 4.3 nM LP-$GCAP1^{CF640R}$ (containing in the aqueous core the equivalent number of $GCAP1^{CF640R}$ molecules present in a 104 µM solution) snapshots were acquired at a 30 min interval, green fluorescence refers to eGFP, red fluorescence refers to LP-$GCAP1^{CF640R}$.
